# Effect of Alkaline-Basic Electrolytes on the Capacitance Performance of Biomass-Derived Carbonaceous Materials

**DOI:** 10.3390/ma13132941

**Published:** 2020-06-30

**Authors:** Boryana Karamanova, Antonia Stoyanova, Maria Shipochka, Svetlana Veleva, Radostina Stoyanova

**Affiliations:** 1 Institute of Electrochemistry and Energy Systems, Bulgarian Academy of Sciences, 1113 Sofia, Bulgaria; boriana.karamanova@iees.bas.bg (B.K.); antonia.stoyanova@iees.bas.bg (A.S.); svetlana_veleva@iees.bas.bg (S.V.); 2 Institute of General and Inorganic Chemistry, Bulgarian Academy of Sciences, 1113 Sofia, Bulgaria; shipochka@svr.igic.bas.bg

**Keywords:** supercapacitors, alkaline electrolytes, biomass-based porous carbons, electrochemical testing, post-mortem analyses

## Abstract

The present work explores in detail the effect of alkaline-basic electrolytes on the capacitance performance of biomass-derived carbonaceous materials used as electrodes in symmetric supercapacitors. The proof-of-concept is demonstrated by two commercial carbon products (YP-50F and YP-80F, Kuraray Europe GmbH, Vantaa, Finland), obtained from coconuts. The capacitance performance of YP-50F and YP-80F was evaluated in three types of basic electrolytes: 6 M LiOH, 6 M NaOH and 6 M KOH. It was found that the capacitance performance of YP-50F improved in the following order: NaOH < LiOH < KOH; Meanwhile, for YP-80F, the order changes to LiOH < NaOH < KOH. After 1000 cycles, the cycling stability of both YP-50F and YP-80F increased in the order NaOH < LiOH < KOH. This order of performance improvement is determined by both the electrolyte conductivity and the interaction between the functional groups of carbonaceous materials and alkaline electrolytes. The reactivity of the functional groups was assessed by postmortem SEM/EDS and X-ray photoelectron spectroscopy (XPS) analyses of the electrodes after prolonged cycling.

## 1. Introduction

Supercapacitors are currently considered as attractive energy storage devices [1]. Having much higher power density than lithium ion batteries and much higher energy densities than conventional capacitors, supercapacitors offer a promising approach to meeting the increasing power demands of energy storage systems. They are a perfect choice for a variety of high power applications thanks to the tunable properties of the electrode materials used for their construction. The ability to design the system according to the expected power/energy profile makes them suitable as alternatives to conventional capacitors and batteries. Electrochemical supercapacitors operate through reversible adsorption of electrolyte counter-ions on the electrode materials [2,3]. Recently, the additional reactions of ion exchange have been demonstrated to take place during supercapacitor function [4]. In general, the storage mechanism remains unclear, since the polarization of the electrode, as well as the compatibility of the electrode materials and electrolyte ions, play crucial roles in determining the electrochemical performance of the supercapacitor [5].

The kind of electrode material used is one of the main factors that determines the supercapacitor performance. The carbon surface chemistry along with the excellent properties of the carbon materials, such as large surface area, highly porous structure, good adsorption properties, and high electrical conductivity, is a very important factor to develop a device with high energy density [2]. Due to the lower specific energy of electrochemical supercapacitors compared to batteries, the demand for new types of electrode materials may reduce the difference between the specific energy of these technologies, and thus, retain the continuity of energy storage and supply [6]. 

Biomass is frequently used for the production of activated carbon (AC), but among many natural organic materials only some of them can be considered as useful precursors [2,7,8,9]. Naturally occurring materials such as rice husk [10], wood [11], cotton [12], bamboo [13], etc. have been more widely used as electrode materials for EC applications in recent years. Biomass-based micro- and nano-porous carbons have also been investigated as active materials in the fabrication of electrodes for ECs [12,14]. These carbons have extremely high specific surface area and specific capacitance as electrode materials for EC applications. The specific feature of biomass-derived carbonaceous materials is the existence of variety of functional groups that can participate in reversible redox reactions in addition to the well-accepted counter-ion adsorption, thus contributing to the storage of extra charges during supercapacitor function [15,16].

Electrolytes are identified as some of the most powerful components in the performance of supercapacitors. Three types of electrolytes can be outlined: aqueous, organic and ionic liquids. As regards carbonaceous electrode materials, aqueous electrolytes ensure an achievement of higher power density than organic-based and ionic liquid electrolytes at the expense of lower energy density [17,18]. The currently tested electrochemical capacitors with aqueous electrolytes compared to the other electrolyte systems provide additional benefits such as low cost, safety, environmental friendliness. Aqueous electrolytes, in their turn, can be divided in acidic, basic and neutral ones. In acidic and basic electrolytes, the working potential window is limited up to 1.2 V, while in the neutral electrolytes the window is extended above 1.2 V reaching a value of 2.2 V. The specific capacitance of carbonaceous electrode materials decreases in the order from acidic and basic to neutral electrolytes [19,20,21,22,23]. The effect of the interaction between the electrodes and electrolytes is also important and very difficult to monitor experimentally. In aqueous electrolytes, the biomass-derived carbonaceous materials undergo surface transformation due to the protonation or deprotonation of the functional groups. Despite the considerable achievements in this area, some difficulties still exist, such as lower energy density and narrow potential window in water electrolytes [24].

The present work aims to explore in detail the effect of alkaline basic electrolytes on the capacitance performance of biomass-derived carbonaceous material. Among the variety of carbonaceous materials, two commercial carbon products (YP-50F and YP-80F, Kuraray Europe GmbH, Vantaa, Finland), obtained from coconuts, are selected since one of them (YP-50F) having more acidic groups and narrow pore size distribution has been shown to exhibit an excellent cycling stability in 6M KOH electrolyte [25]. The storage performance of YP-50F and YP-80F is evaluated in three types of basic electrolytes: 6 M LiOH, 6 M NaOH and 6 M KOH. The effect of the interaction between the YP-50F or YP-80F electrodes and Li^+^, Na^+^ and K^+^ from the basic electrolytes is discussed, based on the results of the post-mortem SEM/EDS and X-ray photoelectron spectroscopy (XPS) analyses of the electrodes after prolonged cell cycling. The established relations between carbon electrode structures and electrolyte composition will help to understand their impact on the storage properties of biomass-based carbonaceous electrodes in symmetric supercapacitors.

## 2. Materials and Methods 

Two activated carbons (YP-50F and YP-80F, Kuraray Europe GmbH, Vantaa, Finland) are the subject of this study. The texture characteristics of YP-50F and YP-80F are summarized in Table S1 (Supplementary Materials). Both YP-50F and YP-80F exhibit specific surface area higher than 1700 m^2^/g, which is a result from the occurrence of micropores. For YP-50F, the average pore diameter is 1.8 nm with a standard deviation of less than 1 nm, while for YP-80F the pores are centred around 2.1 nm with an asymmetric tail between 2 and 6 nm (Figure S1, Supplementary Materials). Detailed texture characterization of two activated carbons is given elsewhere [24].

Biomass-derived YP-50F and YP-80F were analysed by galvanostatic experiments. The supercapacitor cell comprised two identical electrodes containing activated carbon (80 wt.%), graphite ABG 1005 EG-1 (10 wt.%) and polytetrafluoroethylene binder (PTFE, 10 wt.%). The electrodes were assembled in a cell using Viledon 700/18F separator (Freudenberg Filtration Technologies, Milano, Italy) and basic electrolyte: 6 M LiOH, 6 M NaOH or 6 M KOH. The charge-discharge cycling tests were performed using an Arbin Instrument System BT-2000 (American Laboratory Trading, East Lyme, CT, USA). The supercapacitor cells were cycled between 0.01 and 1.2 V at a current load increasing stepwise from 30 to 900 mAg^−1^ for 25 cycles per step. Selected cells were charged/discharged to up to 1000 cycles at a current rate of 60, 240, 360, 600 and 900 mAg^−1^.

The specific discharge capacitance (F g^−1^) was calculated by the Equation [26]:

C = (I × Δt)/(m × Δ V)

where I, Δt, m and Δ V are discharge current, discharge time, mass of active material and voltage window, respectively. On the basis of the specific discharge capacitance, the energy density (E) and power density (P) can be expressed as [27]:

E = C ΔV^2^/7.2

P = E/t

In order to get an insight into the surface and bulk of electrodes during cycling, post-mortem XPS and SEM/EDS analyses were also conducted. The XPS probing of the electrode surface was performed on an AXIS Supra electron spectrometer (Kratos Analitycal Ltd. Manchester, UK) with AlKα radiation (1486.6 eV) and a charge neutralisation system. Microscopic monitoring of the electrodes was carried out using a JEOL JSM 6390 scanning electron microscope (JEOL, Peabody, MA, USA), and the chemical composition was evaluated by EDS analysis—Oxford INCA energy dispersive X-ray spectrometer (Oxford Instruments, Abingdon, UK).

## 3. Results and Discussion

### 3.1. Capacitance Performance of Biomass-Derived Carbon

**Figure 1:**
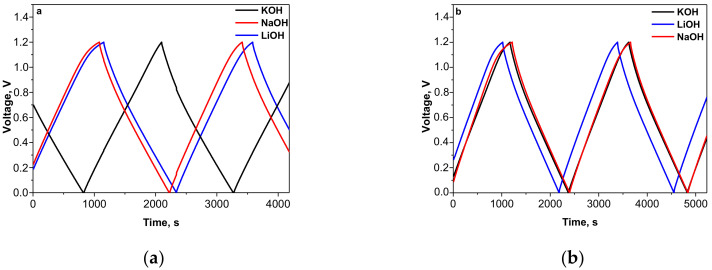
Galvanostatic charge-discharge curves of symmetric supercapacitors with YP-50F (**a**) orYP-80F (**b**) electrodes cycled in basic electrolytes at a current rate of 60 mAg^−1^

Figure 1 compares the charge/discharge curves of carbonaceous electrodes cycled in LiOH, NaOH and KOH electrolytes. All curves exhibit a triangular-shaped profile, which is typical for the capacitive storage mechanism (i.e., electrical double-layer). From the charge–discharge curves, the current resistance of the iR-drop was calculated and corresponding values are presented in Table 1. Comparing the experimental data for the electrode materials under test it can be seen that YP-50F tends to display a slightly lower iR- drop than YP-80F. As regards the electrolyte composition, the iR-drop is the smallest in KOH electrolyte, while the highest value is recorded in NaOH. The magnitude of the iR-drop in LiOH has a medium value between those for KOH and NaOH electrolytes.

**Figure 2:**
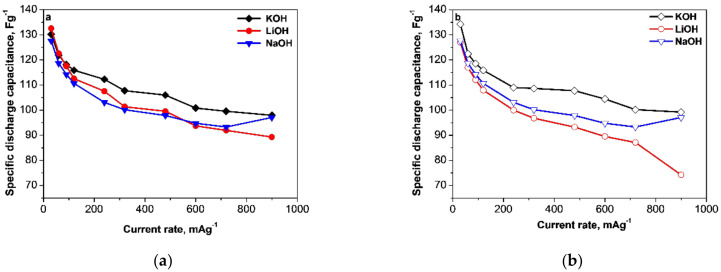
Discharge capacitance depending on the current load for supercapacitors with YP-50F (**a**) or YP-80F (**b**) electrodes in different electrolytes.

The dependences of the capacitance of electrodes on current load are represented graphically in Figure 2. As can be seen, for both YP-50F and YP-80F the capacitance varies between 90 and 130 Fg^−1^ and the highest discharge capacitance within the whole current range is achieved in 6 M KOH. This is the case with the smallest iR-drop (Table 1). The close specific capacitance values of YP-50F and YP-80F can be associated to their porosity (Table S1, Supplementary Materials). Although the average pores diameter is close for both YP-50F and YP-80F, the YP-50F material features narrower pores size distribution than that of YP-80F (Table S1, Supplementary Materials). It is of importance that all pores have dimensions higher than 1 nm, i.e., they are larger in size than the hydrated alkali ions (Table 1). This means that all pores of YP-50F and YP-80F are accessible by alkali ions, as a result of which YP-50F and YP-80F exhibit similar specific capacitance values. It is worth mentioning that a lack of dependence between pore size and capacitance has also been found for carbon monoliths working in electrolyte (C2H5)4NBF4/acetonitrile [28,29]. The physical meaning of this fact is currently unclear.

Furthermore, the data in Figure 2 show also that the electrochemical performance of the two carbons under test is different in LiOH, NaOH and KOH electrolytes, especially at higher current loads. The performance of YP-50F in LiOH and NaOH electrolytes is nearly the same and it is always worse than in KOH. For YP-80F, the performance becomes better following the order LiOH < NaOH < KOH. It is worth mentioning that at the highest current load (i.e., 900 mA.g^−1^), the electrode performance in KOH and NaOH outperforms that in LiOH irrespective of the type of carbon material used.

**Figure 3:**
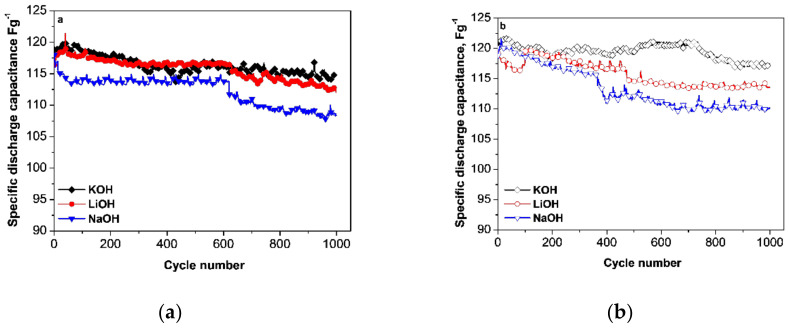
Cycling stability of the discharge capacitance for supercapacitors with YP-50F (**a**) and YP-80F (**b**) electrodes cycled in different electrolytes at a current rate of 60 mAg^−1^

The cycling performance characteristics of YP-50F and YP-80F electrodes in basic electrolytes were further differentiated on the basis of the capacitance stability during cycling (Figure 3). The employed protocol of electrochemical testing includes 1000 charge-discharge cycles in LiOH, NaOH or KOH electrolytes at a current load of 60 mAg^−1^. Although by this protocol, the differences in performance of YP-50F and YP-80F were not distinguishable, the electrolyte composition had a direct impact on the capacitance performance of the biomass-derived carbons. After 1000 cycles, the best performance for both YP-50F and YP-80F electrodes was observed when they were cycled in a KOH electrolyte; the capacitance varied around 110–115 Fg^−1^, with a cycling stability greater than 96%. In the NaOH electrolyte, YP-50F and YP-80F exhibited lower capacitance after 1000 cycles, as well as poorer cycling stability (around 91–92%). An interesting finding was that the performance of both YP-50F and YP-80F in the LiOH electrolyte was intermediate, i.e., between those observed in the KOH and NaOH electrolytes. The capacitance stability of YP-50F and YP-80F upon cycling correlated with the iR-drop values determined from the charge/discharge curves (Table 1). The cycling stability follows the order NaOH < LiOH < KOH.

At first glance, the improved capacitance performance of YP-50F and YP-80F is a simple consequence of the ionic conductivity of basic electrolytes (Table 1). The higher the electrolyte conductivity, the lower the equivalent series resistance (ESR), resulting in high power density for supercapacitors [24]. In aqueous media, the alkali ions are strongly hydrated and their ion-hydrated complex sizes, increasing in the order K^+^ < Na^+^ < Li^+^ (Table 1). As a result, the mobility of alkali metal ions rises in the order of Li^+^ < Na^+^ < K^+^. This is in agreement with the best capacitance performance of carbon-based electrodes in the KOH electrolyte. However, the lower electrode performance in NaOH compared to that in LiOH indicates that the electrolyte conductivity is not the only factor responsible for the charge storage. Fic et al. [23] modelled the ion shape and dimensions in aqueous electrolytes and showed that the poor mobility of Li^+^ ions is more favorable to increasing the overall performance of pseudocapacitive materials (Table 1). In alkaline media, pseudocapacitance reactions depend on the carbonyl functional groups appearing on carbonaceous materials [30]. Recently, we demonstrated that YP-50F and YP-80F have a basic character (see Table S1), but also contain acidic functional groups such as phenolic, carbonyl and carboxylic groups [25]. It appears that the capacitance performance of YP-50F and YP-80F in basic electrolytes is determined by both the electrolyte conductivity and the functional groups of carbonaceous electrodes.

**Figure 4:**
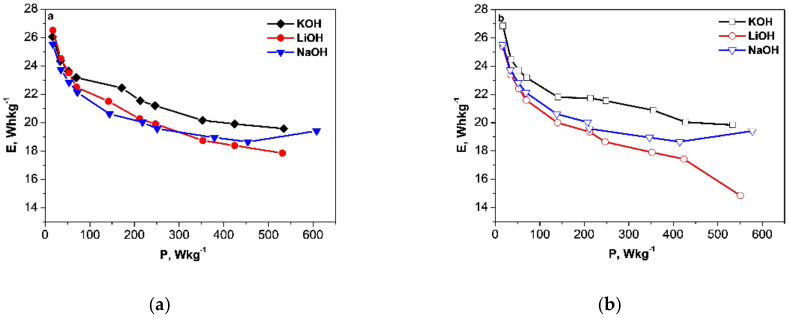
Ragone plots of energy density versus power density for symmetric supercacapacitors with YP-50F (**a**) and YP-80F (**b**) electrodes.

To evaluate the electrochemical performance of activated carbons, Figure 4 shows the Ragone plots for the supercapacitor cells in LiOH, NaOH and KOH electrolytes. The data in the figure show that both materials delivered a similar and high specific energy density of 23–24 Whkg^−1^ at a power density of 50 Wkg^−1^, and maintained a relatively high energy density of 18–21 Whkg^−1^, even at a high power density of 450 Wkg^−1^ in all three electrolytes. For comparison, the ACs obtained from corn husks exhibited a similar energy density (i.e., 21 Whkg^−1^) at a power density of 875 Wkg^−1^ when they were applied as electrodes in supercapacitors with a Na_2_SO_4_ aqueous electrolyte [31]. It is worth noting that, nowadays, commercially available supercapacitors deliver an energy density of less than 10 Whkg^−1^. All these data suggest a good capacitance performance of biomass-derived ACs.

For YP-80F, the specific energy density decreased in the following order: KOH > NaOH > LiOH. This order was consistent with the progressive change in the electrolyte conductivity. For YP-50F, the specific energy density was higher in the KOH electrolyte, while in the NaOH and LiOH electrolytes, the specific energy values were close. This implies that the contribution of pseudocapacitive to the charge storage in YP-50F was more significant in the NaOH and LiOH electrolytes than in KOH.

### 3.2. Postmortem Characterization of Carbon Electrodes

To evaluate the effect of the electrolyte on the supercapacitor properties, XPS and SEM/EDS measurements were taken. The subjects of study were YP-50F and YP-80F electrodes after 1000 cycles in KOH, NaOH or LiOH electrolytes. Although XPS analysis gives information on the outermost surface layers (i.e., up to 5 nm depth), the SEM/EDS is a more “bulky” method (more than 500 nm). Thus, the complementary use of XPS and SEM/EDS measurements allowed us to understand the distribution of chemical components across different depths of the electrode. It should be taken into account, however, that light elements (such as lithium) cannot be detected by EDS analysis, while they are accessible by XPS.

**Figure 5:**
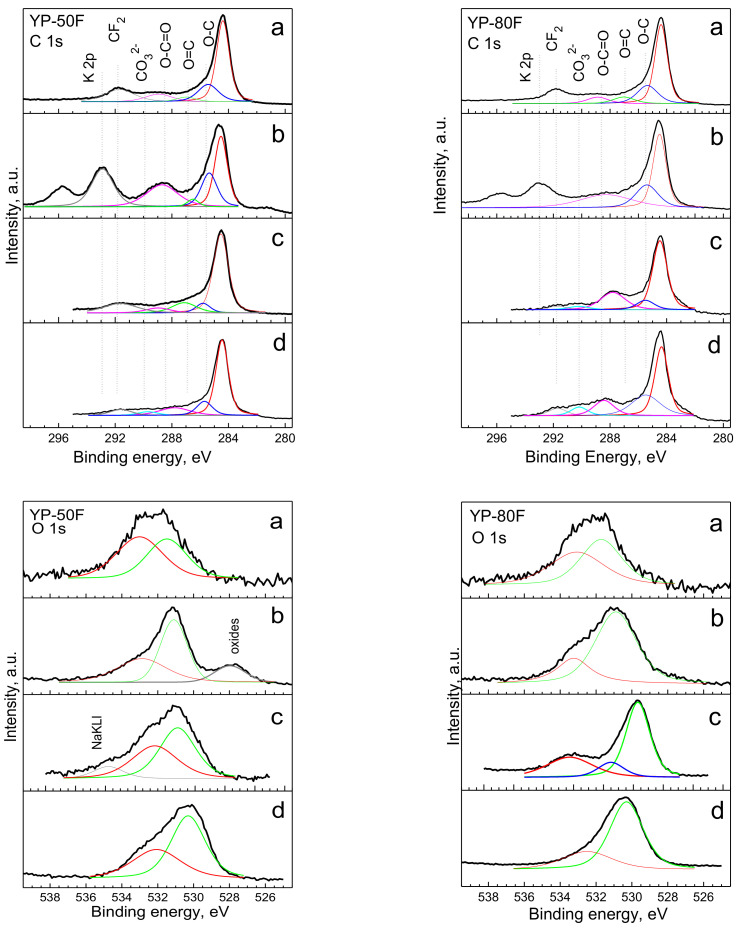
C1s and O1s spectra for YP-50F and YP-80F: pristine YP-50F and YP-80F electrodes (**a**) and cycled YP-50F and YP-80F electrodes in 6M KOH (**b**), 6 M NaOH (**c**) and 6 M LiOH (**d**) electrolytes.

The XPS spectra in the energy region of C 1s and O 1s are presented in Figure 5. For pristine YP-50F and YP-80F, the C1s XPS spectra displayed strong signals due to sp^2^-hybridized graphitic carbon (C-C groups at 284.8 eV). Moreover, there were three additional peaks with a centre of gravity at 285.5 eV, 287.0 eV and 288.8 eV. Taking into account the previously obtained XPS data on carbonaceous materials [32,33], the three peaks can be assumed to come from carbon atoms bonded to oxygens in hydroxyl groups (C-O), carbonyl groups (C=O) and carboxyl groups (COOH). Compared to the C1s spectra, the O1s XPS spectra consisted of two overlapped peaks at 531.5 and 533.0 eV. Irrespective of the uncertainty of the assignments of O1s peaks [34,35], it is reasonable to associate the peak at 531.5 eV to O atoms that are double-bonded in esters, carbonates and acids, while the single bonded O atoms in ketones, ethers and alcohols were likely responsible for the occurrence of the peak at 533.0 eV. It worthy of note that pristine YP-50F and YP-80F were comparable with respect to their functional groups. 

After the electrochemical reaction, the functional groups of both the YP-50F and YP-80F electrodes underwent different transformations depending on the electrolyte composition. In the KOH electrolyte, the relative part of the carboxylic groups increased dramatically, as evidenced by the C1s and O1s spectra (Figure 5). The surface is enriched with carboxylic groups in the case when the reaction proceeded in the LiOH and NaOH electrolytes. The new feature of electrodes cycled in LiOH and NaOH was the appearance of a broad peak centred at around 291 eV, that could be assigned to the C atom in carbonate ions (CO_3_^2−^). This reaction proceeded more readily in the LiOH electrolyte, as well as with YP-80F compared to YP-50F. The XPS data clearly shows that the functional groups of YP-50F and YP-80F interacted with alkaline electrolytes. It is well accepted that hydroxyl and carboxyl functional groups are deprotonated in alkaline media, while the carbonyl groups contribute to the pseudocapacitance reactions via interaction with basic electrolytes [30,36].

**Figure 6:**
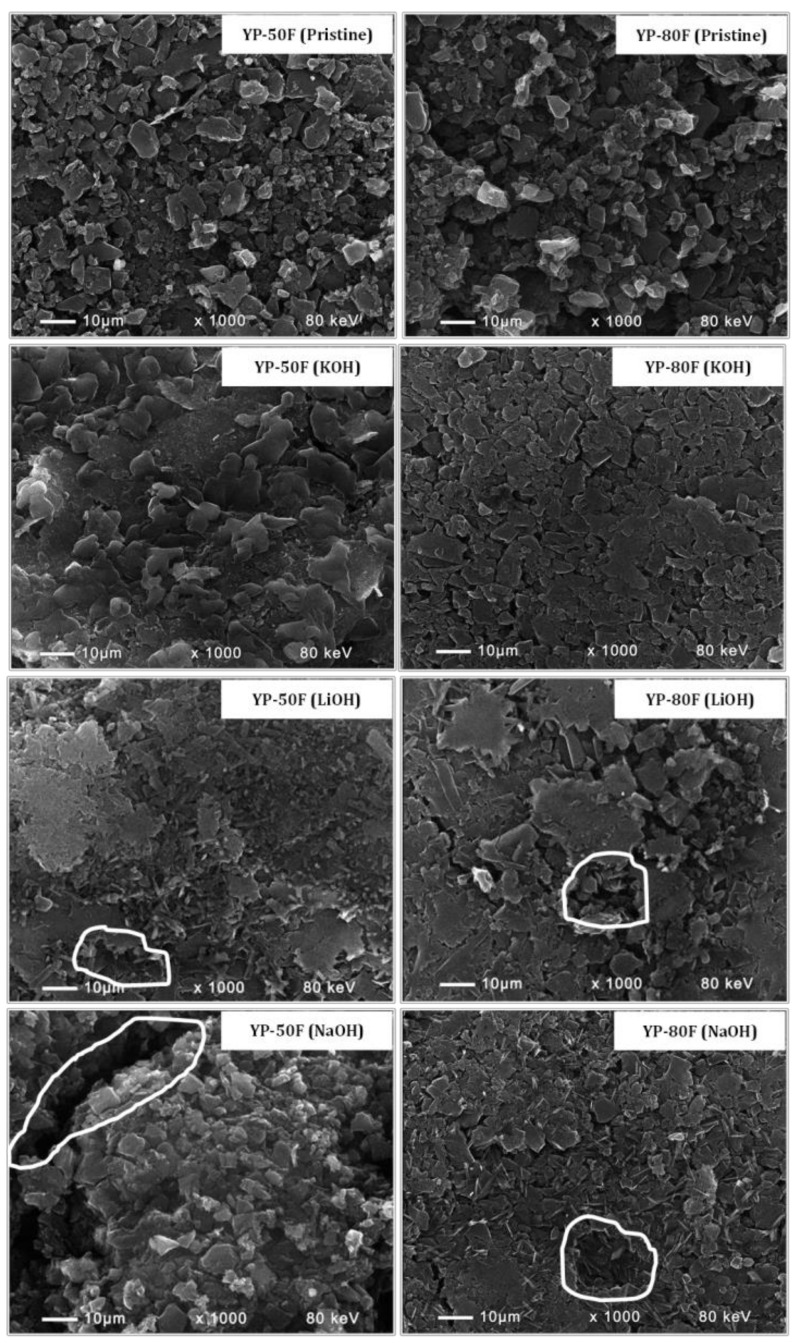
SEM images of pristine electrodes, and after electrochemical tests in KOH, LiOH, NaOH electrolytes. The white lines highlight the development of cracks and faults on the electrode.

The reactivity of the functional groups towards alkaline electrolytes provokes a corresponding change in the electrode morphology (Figure 6). The SEM images of pristine YP-50F and YP-80F electrodes show the formation of a relatively homogeneous and dense surface. After 1000 cycles in alkaline electrolytes, the electrode roughness decreased, with this decrease being dependent on the electrolyte composition. In the KOH electrolyte, the electrode surface became smoother, especially for YP-80F. A smoothing of the electrode surface was also observed when the reaction proceeded in the LiOH electrolyte. However, a closer inspection revealed some cracks. In the NaOH electrolyte, besides cracks, faults also occurred, together with electrode smoothing. It should be noted that the faults were well expressed for YP-50F. The electrolyte-induced change in the electrode morphology may be related to an interaction between functional groups and the alkaline media. The enrichment of the electrode surface with carboxylic groups gave rise to electrode smoothing, while the deposition of carbonate groups caused cracks and faults to occur, which, in turn, induced particle disintegration, leading to the deterioration of the cycling performance, as was experimentally observed in Figure 3. The greater the extent of the faults, the lower the capacitance performance; this is consistent with the observed worse capacitance performance of YP-50F in NaOH compared to LiOH, despite the higher conductivity of NaOH compared to LiOH. 

**Figure 7:**
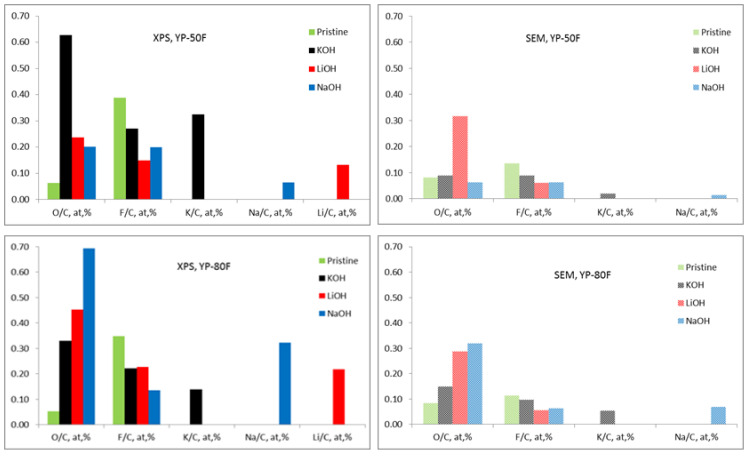
Element ratio (to carbon, at.%) for carbonaceous electrodes calculated from XPS and SEM/EDS data for pristine and worked electrodes in different electrolytes after 1000 cycles. (The origin of F is associated with the Teflon binding agent used. The amount of Li cannot be determined by SEM/EDS.).

To verify this hypothesis, Figure 7 presents the element concentration determined by XPS and SEM experiments. A comparative analysis of the data in Figure 7 outlines two issues for YP-50F and YP-80F working in different electrolytes. First, the surfaces of YP-50F and YP-80F become richer on oxygen and the oxygen amount diminishes along the depth of samples. Second, YP-50F easily adsorbed K^+^ from the electrolyte, while Na^+^ and Li^+^ ions were preferentially adsorbed on YP-80F. All these data support, once again, the assumption that functional groups determine the reactivity of YP-50F and YP-80F towards basic electrolytes.

## 4. Conclusions

The functional groups of biomass-derived carbons, in addition to the electrolyte conductivity, were used to determine the capacitance performance of YP-50F and YP-80F electrodes. In LiOH, NaOH and KOH electrolytes, the acidic functional groups of YP-50F and YP-80F were transformed into carboxylic groups during prolonged cycling, thus making the electrode surface smoother. As a result, the best capacitance performance was achieved, for both YP-50F and YP-80F electrodes, in a KOH electrolyte. In addition, the carbonate groups were preferentially deposited on the electrode surface when the reaction proceeded in LiOH or NaOH electrolytes, contributing to the development of cracks and faults inside the electrode. The electrolyte-induced imperfections caused particle disintegration, which, in turn, led to a deterioration in cycling performance. As a result, the capacitance performance of YP-50F in NaOH was worse than that of LiOH, despite the higher conductivity of NaOH than LiOH. These data demonstrate that understanding the interactions between the functional groups of biomass-derived carbons with electrolytes will be of significance in the design of more effective electrode materials.

## Figures and Tables

**Table 1:** Size and conductivity of electrolytic bare and hydrated ions, iR-drop, discharge capacitance and cycling stability (CS) for 1000 cycles of symmetric supercapacitors with YP-50F or YP-80F electrodes.

Type of Ion	Size of Bare Ion (Å) [23]	Size of Hydrated Ions (Å) [23]	Ionic Conductivity (S cm^2^mol^−1^) [23]	YP-50F			YP-80F		
				iR, V	C, Fg^−1^	CS, %	iR, V	C, Fg^−1^	CS, %
Li^+^	0.60	3.82	38.69	0.024	113	95.4	0.028	114	95.0
Na^+^	0.95	3.58	50.11	0.026	108	92.1	0.028	110	91.5
K^+^	1.33	3.31	73.50	0.023	115	96.6	0.026	117	96.6

## Supplementary Materials

The following are available online at https://www.mdpi.com/1996-1944/13/13/2941/s1, Figure S1: The adsorption-desorption isoterms for carbonaceous electrodes, as well as the corresponding pore size distribution curves, Table S1: Specific surface area (SBET), total pore volume (Vt), micropore volume (Vmicro), average pore diameter (Dav), standard deviation for pore size distribution (sd, nm) and surface functional groups, determined by the Böhm method, for carbonaceous electrodes.
