# Supplementary material for: Effect of Alkaline-Basic Electrolytes on the Capacitance Performance of Biomass-Derived Carbonaceous Materials
Source: Materials (Basel). 2020 Jun 30;13(13):2941. doi: 10.3390/ma13132941 (PMC7372366; doi:10.3390/ma13132941)
Supplement: Supplementary file 1 [file materials-13-02941-s001.pdf]

materials

Supplementary Materials: Effect of the Alkaline-Basic Electrolyte on the Capacitance Performance of Biomass Derived Carbonaceous Materials

Boriana Karamanova [1], Antonia Stoyanova [1], Maria Shipochka [2], Svetlana Veleva [1] and Radostina Stoyanova [2,*]

[1] Institute of Electrochemistry and Energy Systems, Bulgarian Academy of Sciences, Sofia, 1113, Bulgaria; boriana.karamanova@iees.bas.bg (B.K.); antonia.stoyanova@iees.bas.bg (A.S.); svetlana_veleva@iees.bas.bg (S.V.)

[2] Institute of General and Inorganic Chemistry, Bulgarian Academy of Sciences, Sofia, 1113, Bulgaria; shipochka@svr.igic.bas.bg

* Correspondence: radstoy@svr.igic.bas.bg; Tel.: +359–2–979–3915

Table S1. Main texture parameters of the used carbons materials: specific surface area (SBET), total pore volume (Vt), micropore volume (Vmicro), average pore diameter (Dav), standard deviation for pore size distribution (sd, nm) and surface functional groups, determined by the Böhm method.

Samples	S_{BET}, m^2g^{-1}	V_t, cm^3g^{-1}	V_{micro}, cm^3g^{-1}	D_{av}, nm	sd, nm	Basic groups mmolg^{-1}	Acidic groups, mmolg^{-1}
YP-50F	1756	0.80	0.62	1.8	≈1	1.1325	0.2612
YP-80F	2385	1.28	0.73	2.1	2–6	1.1246	0.2402

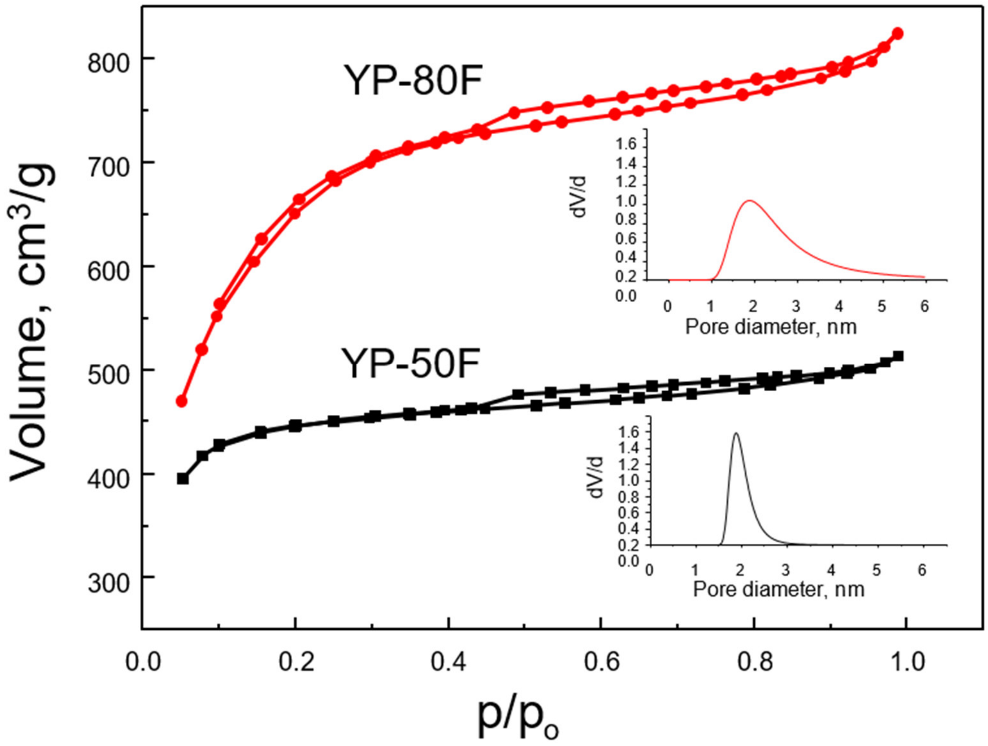

Figure S1. The adsorption-desorption isoterms for carbonaceous electrodes, as well as the corresponding pore size distribution curves.

© 2020 by the authors. Submitted for possible open access publication under the terms and conditions of the Creative Commons Attribution (CC BY) license (http://creativecommons.org/licenses/by/4.0/).
